# Oxygen Consumption Predicts Long-Term Outcome of Patients with Left Ventricular Assist Devices

**DOI:** 10.3390/nu15061543

**Published:** 2023-03-22

**Authors:** Cecilia Veraar, Arabella Fischer, Martin H. Bernardi, Isabella Worf, Mohamed Mouhieddine, Thomas Schlöglhofer, Dominik Wiedemann, Martin Dworschak, Edda Tschernko, Andrea Lassnigg, Michael Hiesmayr

**Affiliations:** 1 Department of Anesthesiology, Intensive Care Medicine and Pain Medicine, Division of Cardiac Thoracic Vascular Anesthesia and Intensive Care Medicine, Medical University of Vienna, 1090 Vienna, Austria; 2 Center for Medical Data Science, Medical University Vienna, 1090 Vienna, Austria; 3 Department of Cardiac Surgery, Medical University of Vienna, 1090 Vienna, Austria; 4 Center for Medical Physics and Biomedical Engineering, Medical University of Vienna, 1090 Vienna, Austria

**Keywords:** left ventricular assist device, oxygen consumption, metabolic and hemodynamic parameters, oxygen delivery, cardiac surgery, intensive care medicine, mitochondrial dysfunction

## Abstract

Reduced oxygen consumption (VO_2_), either due to insufficient oxygen delivery (DO_2_), microcirculatory hypoperfusion and/or mitochondrial dysfunction, has an impact on the adverse short- and long-term survival of patients after cardiac surgery. However, it is still unclear whether VO_2_ remains an efficient predictive marker in a population in which cardiac output (CO) and consequently DO_2_ is determined by a left ventricular assist device (LVAD). We enrolled 93 consecutive patients who received an LVAD with a pulmonary artery catheter in place to monitor CO and venous oxygen saturation. VO_2_ and DO_2_ of in-hospital survivors and non-survivors were calculated over the first 4 days. Furthermore, we plotted receiver-operating curves (ROC) and performed a cox-regression analysis. VO_2_ predicted in-hospital, 1- and 6-year survival with the highest area under the curve of 0.77 (95%CI: 0.6–0.9; *p* = 0.0004). A cut-off value of 210 mL/min VO_2_ stratified patients regarding mortality with a sensitivity of 70% and a specificity of 81%. Reduced VO_2_ was an independent predictor for in-hospital, 1- and 6-year mortality with a hazard ratio of 5.1 (*p* = 0.006), 3.2 (*p* = 0.003) and 1.9 (*p* = 0.0021). In non-survivors, VO_2_ was significantly lower within the first 3 days (*p* = 0.010, *p* < 0.001, *p* < 0.001 and *p* = 0.015); DO_2_ was reduced on days 2 and 3 (*p* = 0.007 and *p* = 0.003). In LVAD patients, impaired VO_2_ impacts short- and long-term outcomes. Perioperative and intensive care medicine must, therefore, shift their focus from solely guaranteeing sufficient oxygen supply to restoring microcirculatory perfusion and mitochondrial functioning.

## 1. Introduction

In patients with end-stage heart failure, heart transplantation (HTX) remains the gold-standard treatment according to European and American guidelines [1,2]. However, the imbalance between the supplement and the demand for allografts remains a bottleneck for clinical transplantation. Therefore, left ventricular assist devices (LVADs) emerged as a life-prolonging alternative for patients with advanced heart failure refractory to medical therapy; either as a destination therapy or as a bridge to candidacy for HTX [3,4]. By increasing cardiac output (CO), the implantation of an LVAD system guarantees sufficient oxygen delivery (DO_2_) as a long-term solution for end-stage heart failure patients with reduced macro-circulation [5]. Maintaining DO_2_ as a surrogate for sufficient macro-circulation and enabling oxygen consumption (VO_2_) as a combined measure of the microcirculatory distribution and mitochondrial activity are cornerstones of modern intensive care medicine [6].

In septic and cardiac arrest patients, mortality is associated with both inadequate DO_2_ due to limited oxygen supply and impaired VO_2_, reflecting mitochondrial dysfunction [6,7,8]. In contrast, in LVAD patients with a constant pump speed to provide a certain CO and, therefore, DO_2_, VO_2_ seems more likely to give us insights into a patient’s physiology [6]. However, in the early postoperative period, hemodynamic instability may even alter the DO_2_ of LVAD patients due to pump settings, hypovolemia, right ventricular dysfunction, ventricular arrhythmia, aortic valve regurgitation and reduced arterial oxygen content (CaO_2_) due to anaemia and/or pulmonary dysfunction [9].

Measuring CO via the pulmonary artery catheter (PAC) has been validated and has been considered to be accurate in both continuous- and pulsatile-flow LVAD patients [10,11]. Continuous CO measurements via the PAC were reported as numerically higher compared to the estimated LVAD pump flow, but within the range [11].

As previously reported, VO_2_ is a measure of microcirculatory perfusion, mitochondrial functioning or insufficient DO_2_ after cardiac surgery that has an impact on the short-term and long-term survival of patients undergoing various cardiac procedures on cardiopulmonary bypass [6,12]. Until now, it remains unknown whether VO_2_ remains an efficient predictor of survival in a population where CO and therefore DO_2_ are supported by a mechanical circulatory support system.

The aim of this study was to determine whether VO_2_ serves as a predictor for short-and long-term on-pump survival and successful bridging to transplantation. Further, we assessed whether VO_2_ remains an independent factor for in-hospital, 1-year and 6-year mortality. Additionally, we compared the longitudinal pattern of PAC-derived metabolic and hemodynamic variables comprising CO, CI, VO_2_ DO_2_, O_2_Extraction Ratio (O_2_ER), mean arterial pressure (MAP) and total peripheral resistance over the first 4 days after LVAD implantation stratified by in-hospital survivors and non-survivors. Lastly, we analyzed whether perioperative VO_2_ levels differ between non-survivors, patients on pump and patients undergoing transplantation after 1 and 5 years.

## 2. Methods and Materials

### 2.1. Ethical Approval

This study was conducted in accordance with the Declaration of Helsinki (as revised in 2013) and was approved by the Ethics Committee of the Medical University of Vienna (EK1099/2022). Data collection was performed in accordance with approved ethical guidelines.

### 2.2. Study Design and Patients

This work was designed as an observational single-center cohort study. We enrolled 93 consecutive patients with terminal heart failure undergoing LVAD implantation with a PAC for hemodynamic monitoring from 2012–2015. Data on survival time was determined in April 2022; data on transplanted patients was determined in 2021. Patient follow-up was censored when patients underwent heart transplantation. The longest follow-up time, either observed or censored, was 6 years. The decision on PAC insertion was based on institutional practice. We included only the first four days of PAC monitoring after surgery and excluded all patients younger than 18 years as well as patients requiring additional right ventricular assist devices (RVAD)s. Since the oxygenator of the temporary RVAD would have biased our measurements. Initial pump settings of the LVAD were determined in the operating room under TEE guidance and reevaluated on a daily basis. PACs were inserted using the Seldinger technique, usually via the right internal jugular approach. Correct positioning with the proximal port located in the SVC and the distal port in the PA was confirmed by X-ray. Calculations of VO_2_, DO_2_ and O_2_ER are shown in Table 1.

### 2.3. Statistical Analysis

Demographic and clinical data were presented using descriptive statistics. The Shapiro–Wilk test was used to examine whether variables were normally distributed. Mean ± standard deviation (SD) and median and interquartile range (25% percentile, 75% percentile) were given for continuous variables. Categorical variables were shown as frequency (percentage). To compare clinical and demographic data we applied the Student’s *t*-test and the Mann–Whitney U test for unpaired normally and non-normally distributed data. Variables such as CO and SvO_2_ were measured continuously using the PAC (CCOmbo, Edwards) and stored in 10 min intervals in the patient data management system (PICIS Critical Care Manager, Barcelona, Spain); VO_2_, O_2_ER and DO_2_ were calculated. CO, SvO_2_, VO_2_, O_2_ER, DO_2_ and hemoglobin (Hb) were averaged for each day for the first 4 days and presented as a median and interquartile range (25% percentile, 75% percentile) for in-hospital survivors and non-survivors. For multiple comparison analyses of CO, SvO_2_, VO_2_, O_2_ER, DO_2_ and Hb over the first 4 days between survivors and non-survivors, we employed the Kruskal–Wallis test. Additionally, we used the Friedman test to analyze the time course of each variable from day 0 until day 4 for survivors and non-survivors.

Furthermore, we applied the Kruskal–Wallis test for the multiple comparisons testing of VO_2_ levels of non-survivors, patients on pump and patients undergoing transplantation after 1 and 5 years. Additionally, we plotted the receiver operating characteristic (ROC) curve and calculated the area under the curve (AUC) for VO_2_, CO, DO_2_ and O_2_ER over the first 4 days and LVAD pump flow after surgery to assess the predictive power for 1-year and 6-year survival and successful transplantation after 5-years. We also performed uni- and multivariate Cox regression analysis for in-hospital, 1- and 6- year mortality. We used the ROC curve-derived cut-offs for VO_2_ and in-hospital survival to stratify patients into two groups. Variables with *p* < 0.05 in the univariate analysis were entered into the multivariate model. For the highest blood lactate, lowest Hb, extracorporeal circulation (ECC) time, and packed red blood cell (PRBC) transfusion during surgery, we used the median value to divide our cohort into two groups. For PRBCs and ECC time we further included a group of missing values. In the model, data is presented as hazard ratio (HR) and 95% confidence interval (CI). All tests were two-sided and *p*-values below 0.05 were considered statistically significant. Statistical analyses were performed using R 3.3.1 and SPSS (version 28.0; IBM SPSS Inc., IL, USA). Figures were plotted using GraphPad Prism (version 8.0; GraphPad Software Inc., CA, USA).

### 2.4. Data Availability

All data generated or analyzed during this study are included in the published article.

## 3. Results

In this single-center cohort study, we included 93 consecutive ICU patients after LVAD implantation. Seventeen patients died during hospitalization. We included 65 patients diagnosed with ischemic cardiomyopathy (CMP), 29 patients with dilated CMP, 1 patient with restrictive CMP and 2 patients with other heart failures. Fifty-three patients received a Heart Ware (Medtronic, MI, USA), 31 patients a HeartMate II (Abbott, Chicago, IL, USA), 8 patients a HeartMate III (Abbott, Chicago, IL, USA) and one patient obtained an MVAD (Medtronic, MI, USA).

Seventeen patients died in the course of their hospitalization; the longest hospital stay was 175 days. A total of 6 patients died within the first 30 days and 28 and 54 patients died within 1 and 6 years after LVAD implantation, respectively. Five and twenty-five patients were successfully transplanted within 1 and 6 years after LVAD implantation. Further details on demographic and clinical data are depicted in Table 2.

### 3.1. Longitudinal Pattern of CI, CO, VO_2_, DO_2,_ O_2_ER, DO_2_ and SvO_2_ of in-Hospital Survivors and Non-Survivors after LVAD Implantation

**Figure 1:**
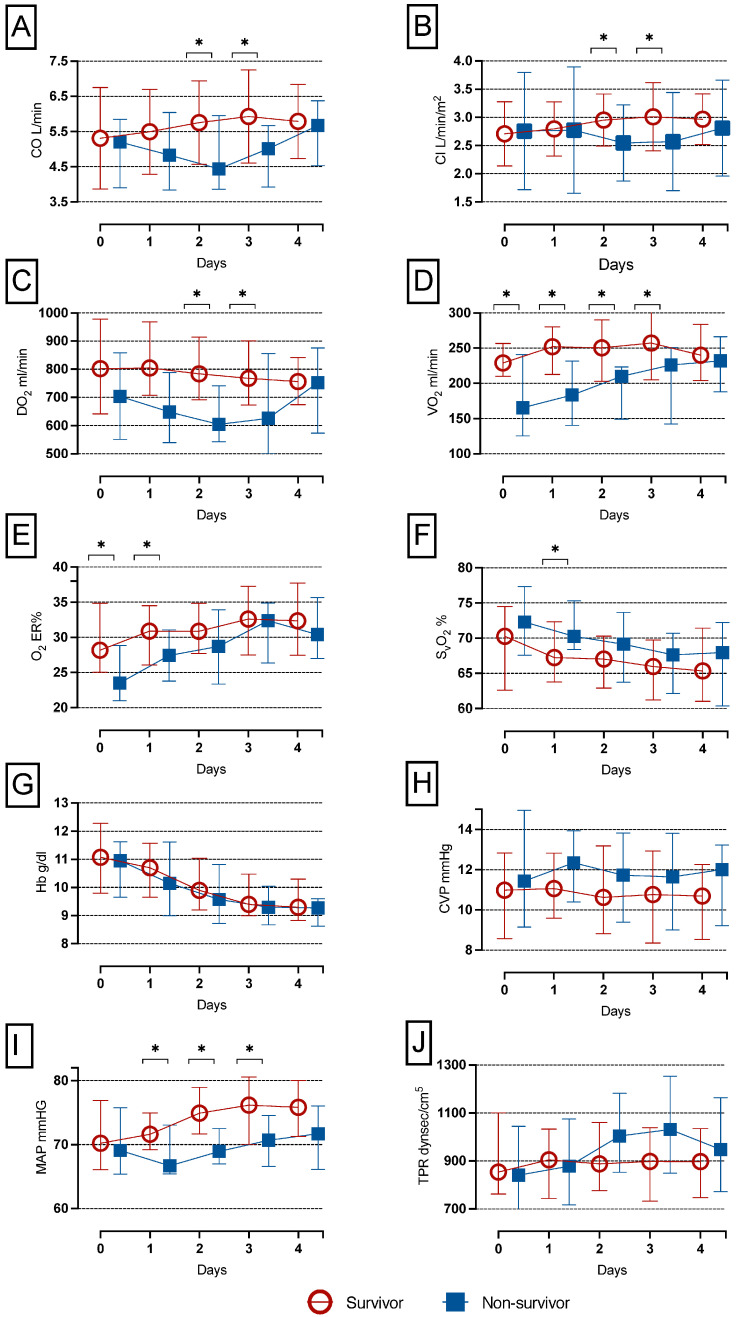
**CO, CI, VO_2_, DO_2_, O_2_ER and SvO_2_ levels of in-hospital survivors and non-survivors after LVAD implantation.** In non-survivors CO, CI and DO_2_ were significantly lower compared to survivors on days 2 and 3 (**A**–**C**). VO_2_ was significantly lower in non-survivors compared to survivors during the first 3 days after surgery (**D**). Non-survivors had significantly lower O_2_ER values on postoperative days 0 and 1 compared to survivors (**E**). SvO_2_ was significantly higher in non-survivors compared to survivors on day 1 (**F**). There was no difference in Hb and CVP between survivors and non-survivors (**G**,**H**). In contrast, there were significantly higher MAP levels in survivors compared to non-survivors from day 1 until day 3 (**I**). TPR did not differ between survivors and non-survivors (**J**). CO, continuous cardiac output; CVP, central venous pressure; DO_2_, oxygen delivery; O_2_ER, oxygen extraction ratio; SvO_2_, mixed venous oxygen saturation; VO_2_, oxygen consumption; * *p* < 0.05.

In non-survivors, CO, CI and DO_2_ were significantly lower compared to survivors on days 2 and 3, but not on days 1 and 4 post LVAD implantation as depicted in Figure 1A–C. In survivors, CO increased significantly from day 0 until days 2, 3 and 4 and also from day 1 until days 3 and 4 ((*p* = 0.001, *p* < 0.001 and *p* < 0.001) and (*p* = 0.001 and *p* = 0.009)). CI rose statistically significantly from day 0 until days 2, 3 and 4 and additionally from day 1 until days 2, 3 and 4 ((*p* = 0.03, *p* < 0.001 and *p* < 0.001) and (*p* = 0.031, *p* = 0.003 and *p* = 0.010)), and DO_2_ decreased statistically significant from day 1 until days 3 and 4 (*p* = 0.009 and *p* = 0.002).

VO_2_ was significantly lower in non-survivors compared to survivors during the first 3 days after surgery, but not on day 4 as shown in Figure 1D. Additionally, VO_2_ increased statistically significantly in survivors from day 0 until days 1, 3 and 4 (*p* = 0.033, *p* < 0.001 and *p* = 0.003).

Non-survivors also had significantly lower O_2_ER values on postoperative days 0 and 1 compared to survivors, but not on days 2, 3 and 4 as depicted in Figure 1F. In addition, there was a significant increase of O_2_ER levels from day 0 until days 3 and 4 in both survivors and non-survivors ((*p* = 0.03 and *p* = 0.02) and (*p* = 0.009 and *p* = 0.009).

SvO_2_ was significantly higher in non-survivors compared to survivors on day 1, but not on days 0, 2,3 and 4 as depicted in Figure 1F. In survivors, there was a significant decrease from day 0 until days 3 and 4 and from day 1 until day 3 (*p* = 0.02, *p* = 0.009 and *p* = 0.17).

In contrast, we did not find significant differences in hemoglobin and central venous pressure (CVP) between survivors and non-survivors as detailed in Figure 1G,H. We observed a significant decrease in Hb levels from day 0 until days 2, 3 and 4 in all patients (*p* = 0.014, *p* < 0.001 and *p* < 0.001). CVP changed neither in survivors nor in non-survivors over time. MAP was significantly higher in survivors compared to non-survivors on days 1, 2 and 3 as depicted in Figure 1I. Furthermore, in survivors, we found a significant increase in MAP from day 0 until days 2, 3 and 4 (*p* < 0.001, *p* < 0.001 and *p* < 0.001). In non-survivors MAP increased significantly from day 0 until days 3 and 4 (*p* = 0.010 and *p* = 0.005). TPR did not differ statistically significant during the first 4 days between survivors and non-survivors as shown in Figure 1J. Additionally, there were no statistically significant changes in TPR over the longitudinal time course of survivors and non-survivors (*p* = 0.250 and *p* = 0.951).

### 3.2. Differences in VO_2_ Levels of Non-Survivors, Patients on Pump and Patients Undergoing Transplantation after 1 and 5 Years

**Figure 2:**
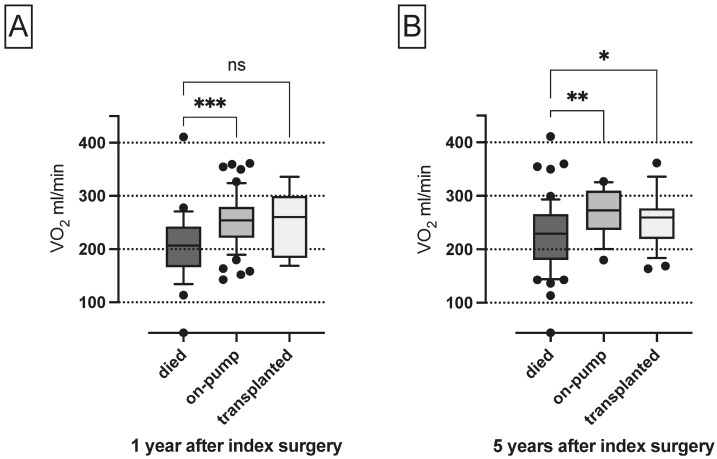
VO_2_ levels during the first 4 days after surgery of 1- and 5-years non-survivors, patients on pump and patients undergoing transplantation. ns, not significant; VO_2_, oxygen consumption; * *p* < 0.05, ** *p* < 0.01, *** *p* < 0.001.

VO_2_ levels during the first 4 days after surgery were significantly lower in patients who died within 1 year compared to on-pump patients, but not compared to patients undergoing transplantation (median 207 mL/min (166, 243), 254 mL/min (221, 279) and 260 mL/min (184, 300); *p* = 0.002), as depicted in Figure 2A.

In contrast, VO2 levels after surgery were significantly lower in 5-year non-survivors compared to patients on pump and patients who were transplanted after 5 years (median 229 mL/min (180, 266), 273 mL/min (236, 309) and 259 mL/min (219, 277); *p* = 0.007), as shown in Figure 2B.

### 3.3. The Association between VO_2_, CO and DO_2_ and Short- and Long-Term Outcomes

**Figure 3:**
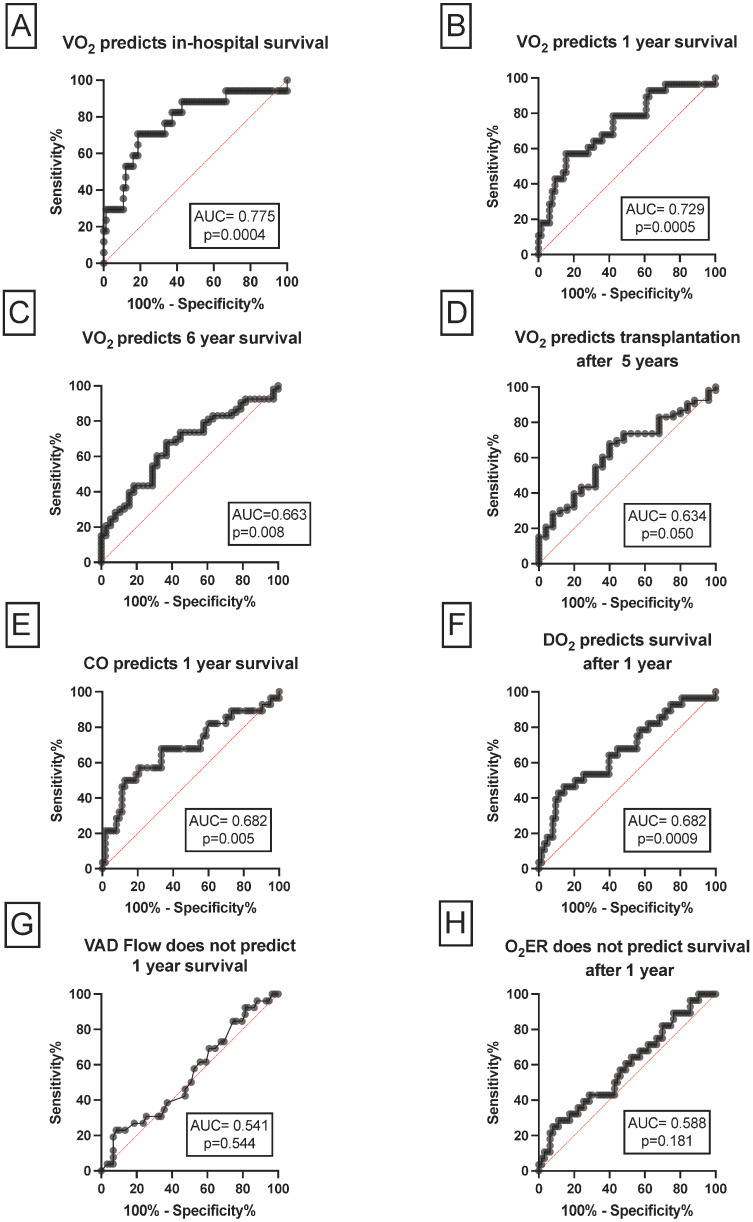
**The association of VO_2_, CO and DO_2_ and short- and long-term outcomes.** Elevated VO_2_ predicted in-hospital, 1-year and 6-year survival and successful transplantation within 5 years (**A**–**D**). Increased CO and DO_2_ levels predicted 1-year survival (**E**,**F**). Increased VAD flow and O_2_ER did not predict 1-year survival (**G**,**H**). AUC, area under the curve, CO, cardiac output; VAD ventricular assist device; VO_2_, oxygen consumption.

Elevated VO_2_ predicted in-hospital survival with an AUC of 0.77 (95% CI: 0.6–0.9; *p* = 0.0004), as shown in Figure 3A. The ROC curve-derived cut-off value for VO_2_ of 210 mL/min had a sensitivity of 70% and a specificity of 81 % to stratify patients regarding in-hospital survival.

As depicted in Figure 3B, increased VO_2_ predicted 1-year survival with an AUC of 0.72 (95% CI: 0.6–0.8; *p* = 0.0005). The ROC curve-derived cut-off value for VO_2_ of 245 mL/min had a sensitivity of 78% and a specificity of 57 % to stratify patients regarding 1-year survival.

Elevated VO_2_ also predicted 6-year survival with an AUC of 0.68 (95% CI: 0.5–0.7; *p* = 0.0081). A cut-off value for VO_2_ of 248 mL/min had a sensitivity of 67% and a specificity of 63 % to stratify patients regarding 6-year survival, as detailed in Figure 3C.

Further, high VO_2_ levels predicted successive transplantation after 5 years with an AUC of 0.63 (95% CI: 0.5–0.7; *p*= 0.050), as pictured in Figure 3D. The ROC curve-derived cut-off value for VO_2_ of 258 mL/min had a sensitivity of 54% and a specificity of 73% to divide our cohort into patients who underwent successful transplantation within 5 years and patients who died within 5 years.

Increased CO levels predicted 1-year survival with an AUC of 0.68 (95 % CI: 0.5–0.8; *p* = 0.005), as depicted in Figure 3E. The ROC curve-derived cut-off value for CO of 5.4 L/min had a sensitivity of 68% and a specificity of 66% regarding 1-year survival.

Similarly, elevated DO_2_ predicted 1-year survival with an AUC of 0.67 (95% CI: 0.5–0.7; *p* = 0.009), as detailed in Figure 3F. The ROC curve-derived cut-off value for DO_2_ of 761 mL/min had a sensitivity of 64% and a specificity of 60% to divide patients into 1-year survivors or non-survivors.

As shown in Figure 3G,H, increased LVAD flow and O_2_ER did not predict 1-year survival. The AUC was 0.54 (95% CI: 0.4–0.6; *p* = 0.544) and 0.58 (95% CI: 0.5–0.7; *p* = 0.181), respectively.

### 3.4. Univariate and Multivariate Cox Regression Analyses for VO_2_ for in-Hospital as well as 1- and 6-Year Mortality

VO_2_ below the ROC curve-derived cut-off of 210 mL/min was associated with increased mortality in the univariate model and remained an independent factor for mortality in the multivariate analysis for in-hospital, 1 and 6 years after LVAD implantation. Age > 75 years was associated with increased in-hospital, 1- and 6-year mortality in the univariate and remained an independent factor for 1-year mortality in the multivariate analysis. sCR > 2.2 mg/dL was associated with increased in-hospital, 1-year and 6-year mortality in the uni- and multivariate analysis. Lactate levels > 3.6 mmol/L had a significant HR in the univariate but not in the multivariate analysis for in-hospital, 1-year and 6-year mortality. Patients with a minimum Hb < 8 mg/dL had a significantly increased in-hospital and 1-year mortality in the univariate but not in the multivariate analysis. Furthermore, patients receiving > 3 PRBCs had a significantly increased HR for in-hospital, 1- and 6-year mortality in the uni- and the multivariate analysis (Table 3).

## 4. Discussion

Increased VO_2,_ over the first 4 days had the highest AUC to predict in-hospital, 1- and 6-year survival. Conversely, impaired VO_2_ remained an independent factor for increased in-hospital, 1- and 6-year mortality after LVAD insertion in the uni- and multivariate model. At POD 0 and 1, VO_2_ but not DO_2_ was significantly lower in non-survivors. In parallel, O_2_ER was significantly decreased and SvO_2_ was significantly higher during the first two days after surgery. These findings suggest that decreased VO_2_ was not induced by limited DO_2_, indicating an uncoupling of macrocirculatory and microcirculatory hemodynamics early after ICU admission. Consequently, restoration of the macrocirculation does not necessarily mean that microvascular perfusion is adequately functioning [13].

Vasoplegia is a common finding after LVAD insertion occurring with a prevalence of up to 33% within 48 h after surgery [14]. In our study, we found lower MAP levels in non-survivors from day 1 until day 3. Therefore, it seems likely that vasoplegia contributed to a compromised microcirculation in non-survivors, leading to shunts within the tissue and consecutively impaired off-loading of Hb-bound oxygen.

Furthermore, altered microcirculatory perfusion has been reported after the institution of a non-pulsatile blood flow [15]. Even though LVAD systems cause stable continuous blood flow in both survivors and non-survivors, pulsatility is especially diminished in non-survivors on full LVAD support with absent aortic valve opening [16].

Ischemia/reperfusion injury during CPB could also explain an initial mitochondrial dysfunction even after adequately restoring the oxygen supply. In contrast to non-survivors, survivors may counteract these changes by early activation of mitochondrial biogenesis [17]. In the literature, this condition is termed “cytopathic hypoxia” denoting a diminished production of adenosine triphosphate (ATP) despite normal oxygen levels within the mitochondria of cells [18].

In contrast, at POD 2 and 3, it seems more likely that diminished VO_2_ was also affected by reduced DO_2_. In LVAD patients, impaired CO and DO_2_ may indicate that these patients were more likely on full LVAD support as a result of a poorer native LV function. Particularly since the goal of LVAD RPM titration in the early postoperative period is the maintenance of right ventricular geometry, avoiding midline shifts and suction, and enabling intermittent aortic valve opening, rather than maximizing LVAD flow [19]. Moreover, we found steadily decreasing Hb levels in both survivors and non-survivors. As in LVAD patients, DO_2_ cannot be enhanced by indiscriminately raising CO, it may be important to target higher Hb levels in critical patients to optimize DO_2_.

Our study has several limitations due to the retrospective analysis of prospectively and automatically collected data. Moreover, we assessed VO_2_ via the PAC and did not use indirect calorimetry, which is known as the gold standard for measuring VO_2_, carbon dioxide production and resting energy expenditure [20,21]. Studies correlating VO_2_ values measured via the PAC in comparison to indirect calorimetry remain outstanding. Assessing VO_2_ via the PAC has the advantage of being able to measure VO_2_ continuously over a prolonged period of time. Furthermore, PAC insertion during LVAD implantation is part of our institutional protocol. Therefore, we could include our patients consecutively, without selection bias. Another limitation of our study concerns varying LVAD systems with different hemodynamic profiles, thus complicating the uniformity of our data. Further, we had no information on whether patients were on full or partial LVAD support and there was limited power to detect differences according to LVAD type. Another weakness of our study concerns interpreting 6-year survival, since up to 2/3 of all survivors were transplanted and only one-third remained on LVAD support. As a result, we additionally plotted the ROC for successful transplantation within 5 years. Furthermore, there is no medically determined cut-off value to define low and high VO_2_. Consequently, we employed the best cut-off determined via the ROC curve for VO_2_ and in-hospital survival to divide patients into two groups to perform the uni- and the multivariate cox regression. Another drawback of our study concerns the suspected mitochondrial dysfunction, which we assumed solely based on the interpretation of metabolic and hemodynamic parameters without performing mitochondrial diagnostic testing. Measuring cell-free mtDNA as a surrogate for mitochondrial functioning remains unexplored in LVAD patients [22].

## 5. Conclusions

Non-survivors after LVAD implantation have a reduced VO_2_ possibly as a result of, microcirculatory hypoperfusion, reduced oxygen supply and mitochondrial dysfunction. Reduced VO_2_ remained an independent factor for increased mortality in the uni- and multivariate model for in-hospital, 1- and 6-year mortality post LVAD implantation. Perioperative and intensive care medicine must therefore shift their focus from solely guaranteeing sufficient oxygen supply to restoring microcirculatory perfusion and mitochondrial functioning.

## Figures and Tables

**Table 1:** Employed formulas for the calculation of venous and arterial oxygen content, oxygen delivery, oxygen consumption, oxygen extraction ratio and total peripheral resistance.

Variables	
CvO_2_	=Hb × 1.37 × S_v_O_2_ + 0.003 × P_v_O_2_
CaO_2_	=Hb × 1.37 × S_a_O_2_ + 0.003 × P_a_O_2_
DO_2_	=CO × C_a_O_2_ × 10
VO_2_	=CO × (C_a_O_2_ − C_v_O_2_) × 10
O_2_ER	=VO_2_/DO_2_ = C_a_O_2_/(C_a_O_2_ − C_v_O_2_)
TPR	=(MAP − CVP)/CO × 80

CaO_2_, arterial oxygen content; CO, cardiac output; CvO_2_, venous oxygen content; CVP, central venous pressure; DO_2_, oxygen delivery; Hb, hemoglobin; MAP, mean arterial pressure; TPR, total peripheral resistance; VO2, oxygen consumption; O_2_ER, oxygen extraction ratio.

**Table 2:** **Demographic and clinical data.** Demographic and clinical data of hospital survivors and non-survivors.

	Total *n* = 93	Survivor *n* = 76	Non-Survivor *n* = 17	*p*-Value
**Demographic data**				
Female: male ^#^	12 (100): 81 (100)	8 (66): 68 (84)	4 (33): 13 (16)	0.148
Age (years) ^##^	61 ± 9	60 ± 9	65 ± 8	**0.041**
BMI (kg/m^2^) ^##^	26.4 ± 4.5	26.5 ± 4.4	26.1 ± 4.8	0.736
BSA (m^2^) ^##^	1.9 ± 0.2	1.9 ± 0.2	1.9 ± 0.3	0.406
DM ^#^	27 (29)	22 (28)	5 (29)	0.970
COPD ^#^	20 (21)	15	5 (29)	0.380
sCR *	1.6 ± 0.9	1.4 ± 0.5	2.3 ± 1.6	**0.016**
**Diagnosis**				
iCMP ^#^	56 (60)	48 (64)	8 (47)	
dCMP ^#^	29 (31)	25 (34)	4 (23)	
iCMP and dCMP ^#^	4 (4)	1 (2)	3 (17)	
rCMP ^#^	1 (3)	-	1 (5)	
other ^#^	2 (2)	1 (2)	1 (6)	**0.004**
**Intermacs level**				
1 ^#^	23 (25)	16 (21)	6 (35)	
2 ^#^	7 (8)	5 (7)	2 (12)	
3 ^#^	36 (39)	30 (40)	6 (35)	
4–7 ^#^	23 (24)	21 (28)	2 (12)	
Missing ^#^	4 (4)	4 (4)	2(6)	0.516
**Device**				
HVAD ^#^	53 (57)	41 (54)	12 (71)	
Heart Mate II ^#^	31 (33)	27 (36)	4 (23)	
Heart Mate III ^#^	8 (9)	7 (9)	1 (6)	
MVAD ^#^	1 (1)	1 (1)	-	0.618
**Device settings after surgery**				
Pulsatility index ^##^	3.2 (2.6, 4,0)	3.4 (3.0, 4.2)	3.0 (2.1, 4.0)	0.220
Flow (l/min) ^##^	4.2 (3.4, 5.0)	4.3 (3.5, 5.0)	4.0 (2.8, 5.0)	0.448
**Perioperative data**				
Lactate max (mmol/L) ^##^	2.8 (2.2, 3.7)	2.6 (2.1, 3.4)	3.6 (2.8, 4.8)	**0.013**
Hb min (g/dl) ^##^	8.8 (8.0, 9.8)	9.0 (8.2, 9.9)	8.4 (7.6–9.4)	0.059
PRBC (count) ^##^	4.0 (2.0, 6.0)	3.5 (2.0, 5.7)	6.0 (4.0, 9.5)	**0.002**
FFP (count) ^##^	4.5 (3.0, 9.0)	3.5 (3.0, 8.7)	7.0 (3.0, 10.0)	0.295
SDP (count) ^##^	2.0 (1.0, 2.2)	2.0 (1.0, 2.0)	3.0 (2.0, 4.0)	**0.001**
ECC time (min) ^##^	105 (69, 149)	104 (65, 152)	109 (99, 148)	0.618
Anesthesia time (min) ^##^	370 (320, 467)	370 (320, 458)	411 (320, 537)	0.345
**Hemodynamic parameter**				
VO_2_ overall (ml/min) *	240 (198, 274)	251 (218, 276)	188 (143, 231)	**<0.001**
CO overall (L/min) *	5.5 (4.6, 6.2)	5.6 (5.0, 6.4)	4.4 (3.9, 6.0)	**0.019**
CI overall (L/min/m^2^) *	2.8 (2.4, 3.0)	2.8 (2.6, 3.1)	2.4 (2.1, 2.8)	**0.009**
DO_2_ overall (ml/min) *	767 (649, 864)	789 (665, 879)	610 (545, 806)	**0.019**
O_2_ER overall (%) *	30.8 (27.3, 34.6)	31.5 (27.6, 35.1)	30.1 (24.0, 32.4)	0.091
SvO_2_ overall (%) *	67 (63, 72)	67 (63, 71)	69 (66, 74)	0.104
CVP overall (mmHg) *	11 (9, 13)	11 (9, 12)	11 (9, 13)	0.298

BMI, body mass index; COPD, chronic obstructive pulmonary disease; dCMP, dilated cardiomyopathy; DM, diabetes mellitus; DO_2_, oxygen delivery; ECC, extracorporeal circulation; FFP, fresh frozen plasma; Hb, hemoglobin; HTX, heart transplantation; IQR interquartile range; iCMP, ischemic cardiomyopathy; LVAD, left ventricular assist device; n, number; O_2_ER, oxygen extraction ratio; PRBC, packed red blood cells; rCMP, restrictive myopathy; sCR, serum creatinine; SD, standard deviation; SDP, single donor platelets; VO2, oxygen consumption, ^#^ n (%),^##^ mean ± SD, * median (IQR).

**Table 3:** **Uni- and multivariate cox regression analysis for in-hospital, 1 and 6-year mortality after LVAD Implantation.** The univariate model was performed for demographic and perioperative characteristics. The multivariate model included only statistically significant categories of the univariate model.

Cox Regression Analysis							
		Univariate Model			Multivariate Model		
		HR	CI 95%	*p*-Value	HR	CI 95%	*p*-Value
	**In-hospital mortality**						
**VO_2_**	>210 mL/min ^#^	1.0			1.0		
	≤210 mL/min	**7.1**	2.5–20.3	**<0.001**	**15.0**	2.3–95.4	**0.004**
**Gender**	male ^#^	1.0					
	female	2.0	0.6–6.3	0.205			
**Age**	<55 years ^#^	1.0			1.0		
	55–65 years	2.6	0.5–12.4	0.218	3.8	0.5–19.2	0.193
	66–75 years	2.5	0.4–12.9	0.270	0.4	0.0–3.2	0.468
	>75 years	**11.0**	1.5–78.7	**0.017**	282	0.0–2785	0.901
**BMI**	<25 kg/m^2 #^	1.0					
	25–30 kg/m^2^	0.8	0.2–2.4	0.761			
	>30 kg/m^2^	0.4	0.0–2.0	0.284			
**sCr**	≤1.2 mg/dL ^#^	1.0					
	1.2–2.2 mg/dL	2.2	0.5–8.4	0.236	**21.4**	2.7–165	**0.003**
	>2.2 mg/dL	**7.9**	1.9–31.9	**0.003**	**30.4**	4.5–204	**<0.001**
**ECC time**	≤170 min ^#^	1.0					
	>170 min	0.8	0.1–6.8	0.873			
	off-pump	1.6	0.6–4.5	0.296			
**Hb min**	≥8 g/dL ^#^	1.0			1.0		
	<8 g/dl	**2.8**	1.0–7.4	**0.034**	2.1	0.6–7.0	0.193
**Lac max**	≤3.6 mmol/L^#^	1.0			1.0		
	>3.6 mmol/L	**3.9**	1.5–10.3	**0.005**	1.1	0.3–3.8	0.768
**PRBCs**	≤3 units	1.0					
	>3 units	**12.4**	1.6–94.6	**0.015**	427	0.0–4130	0.925
	no PRBCs	1.5	0.0–24.6	0.760	**0.0**	0.02–1.0	**0.019**
**CVP**	≤11.1 mmHg	1.0					
	>11.1 mmHg	2.5	0.9–7.0	0.061			
	**1-year all-cause mortality**						
**VO_2_**	>210 mL/min ^#^	1.0			1.0		
	≤210 mL/min	**4.4**	2.0–5.7	**<0.001**	**3.4**	1.4–8.1	**0.005**
**Gender**	male ^#^	1.0					
	female	1.1	0.4–3.3	0.776			
**Age**	<55 years ^#^	1.0			1.0		
	55–65 years	**3.5**	1.0–12.1	0.094	3.0	0.8–11.1	0.164
	66–75 years	2.8	0.7–10.7	0.123	1.0	0.5–8.6	0.261
	>75 years	**9.0**	1.4–54.3	**0.016**	**22.8**	2.1–246	**0.010**
**BMI**	<25 kg/m^2 #^	1.0					
	25–30 kg/m^2^	1.0	0.4–2.3	0.955			
	>30 kg/m^2^	0.5	0.1–1.6	0. 292			
**sCr**	≤1.2 mg/dL ^#^	1.0			1.0		
	1.2–2.2 mg/dL	1.3	0.5–3.2	0.490	**3.4**	1.2–9.6	**0.018**
	>2.2 mg/dL	**3.9**	1.4–10.8	**0.009**	**10.8**	3.0–28.1	**<0.001**
**ECC time**	≤170 min ^#^	1.0					
	>170 min	1.3	0.3–4.5	0.679			
	off-pump	1.0	0.4–2.2	0.979			
**Hb min**	≥8 g/dL ^#^	1.0			1.0		
	<8 g/dL	**2.4**	1.1–5.2	**0.025**	1.7	1.2–12.2	0.195
**Lac max**	≤3.6 mmol/L ^#^	1.0			1.0		
	>3.6 mmol/L	**2.5**	1.2–5.4	**0.012**	1.2	0.5–2.9	0.650
**PRBCs**	≤3 units	1.0			1.0		
	>3 units	**3.6**	1.3–9.7	**0.010**	**3.9**	1.2–12.2	**0.019**
	no PRBCs	0.9	0.2–3.9	0.940	0.8	0.1–5.6	0.859
**CVP**	≤11.1 mmHg	1.0					
	>11.1 mmHg	1.6	0.8–3.5	0.169			
**6-year all-cause mortality**							
**VO_2_**	>210 mL/min ^#^	1.0					
	≤210 mL/min	**2.5**	1.4–4.4	**<0.001**	**3.0**	1.1–3.9	**0.022**
**Gender**	male ^#^	1.0					
	female	0.9	0.4–2.1	0.967			
**Age**	<55 years ^#^	1.0			1.0		
	55–65 years	1.8	0.9–3.9	0.092	0.5	0.2–1.2	0.155
	66–75 years	**2.2**	1.0–4.7	**0.046**	0.6	0.2–1.3	0.219
	>75 years	3.0	0.6–14.1	0.147	2.4	0.4–12.4	0.271
**BMI**	<25 kg/m^2 #^	1.0					
	25–30 kg/m^2^	0.9	0.4–1.7	0.784			
	>30 kg/m^2^	1.1	0.5–2.2	0.673			
**sCr**	≤1.2 mg/dL ^#^	1.0			1.0		
	1.2–2.2 mg/dL	1.4	0.7–2.6	0.280	**2.2**	1.1–4.5	**0.022**
	>2.2 mg/dL	**3.3**	1.5–7.4	**0.003**	**7.7**	2.8–21.5	**<0.001**
**ECC time**	≤170 min ^#^	1.0					
	>170 min	1.1	0.4–3.0	0.756			
	off pump	0.9	0.5–1.7	0.942			
**Hb min**	≥8 g/dL ^#^	1.0					
	<8 g/dl	1.7	0.9–3.1	0.081			
**Lac max**	≤3.6 mmol/L ^#^	1.0					
	>3.6 mmol/L	**1.7**	1.0–3.1	**0.042**	1.2	0.6–2.4	0.539
**PRBCs**	≤3 units	1.0					
	>3 units	**2.3**	1.2–4.5	**0.014**	**2.9**	0.9–4.0	**0.008**
	no PRBCs	1.3	0.6–3.0	0.434	1.7	0.6–4.4	0.272
**CVP**	≤11.1 mmHg	1.0					
	>11.1 mmHg	1.4	0.8–2.5	0.157			

Significant *p*-values are written in bold. BMI, body mass index; CI, confidence interval; d, days; ECC, extracorporeal circulation; FFP, fresh frozen plasma; Hb, hemoglobin; HR, hazard ratio; PRBC, packed red blood cells; sCR, serum creatinine; ^#^ Reference.

## Data Availability

All data generated or analyzed during this study are included in this published article.
